# Supplementary material for: Comparative multiomics analysis of cell physiological state after culture in a basket bioreactor
Source: Sci Rep. 2022 Nov 23;12:20161. doi: 10.1038/s41598-022-24687-4 (PMC9686226; doi:10.1038/s41598-022-24687-4)

_023666045.1:2843125:2843251:+@NW_023666045.1:2843808:2848428:+@NW_023666045.1:2844576:284842

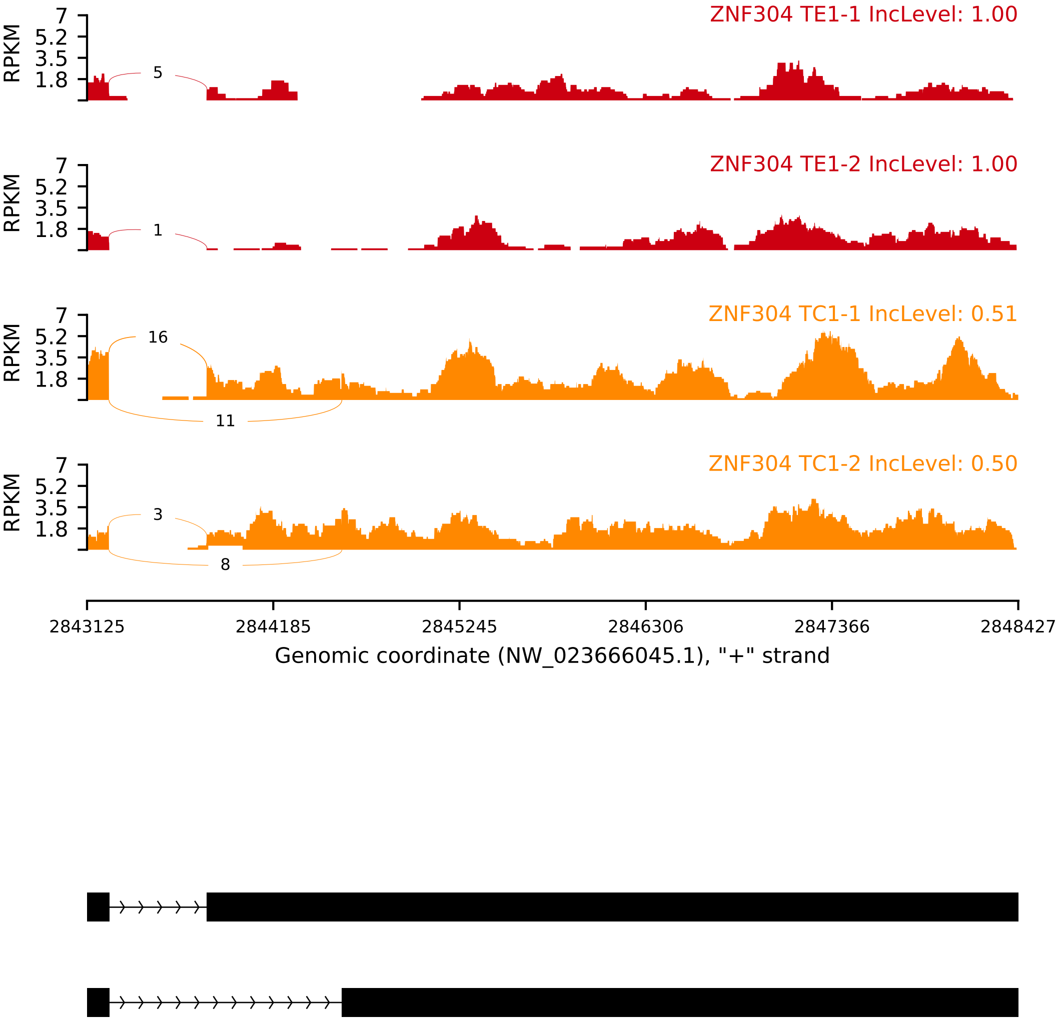

Supplement: Supplementary file 1 — Supplementary Information 1. [file 41598_2022_24687_MOESM1_ESM.zip › raw data/Transcriptomic raw data/7.AS/2.dasplot/TE1vsTC1/A3SS/7_ZNF304_NW_023666045.1_2843125_2843251_+@NW_023666045.1_2843808_2848428_+@NW_023666045.1_2844576_2848428_+.pdf]
